# Supplementary material for: Age Specific Risks of Uterine Cancer in Type 2 Diabetes and Associated Comorbidities in Taiwan
Source: Cancers (Basel). 2022 Oct 7;14(19):4912. doi: 10.3390/cancers14194912 (PMC9564306; doi:10.3390/cancers14194912)
Supplement: Supplementary file 1 [file cancers-14-04912-s001.zip › cancers-1882110-final-suppl.pdf]

Supplementary Materials: Age Specific Risks of Uterine Cancer in Type 2 Diabetes and Associated Comorbidities in Taiwan

Hui-Shan Liu, Chin-Der Chen, Chung-Chen Lee, Yong-Chen Chen and Wen-Fang Cheng

Table S1. Coding diagnosis of any cancer, hysterectomy, comorbidities, and drug use in enrolled subjects.

	Definitions	Code
**Exclusion**		
Diagnosis of any cancer	Defined from diagnosis[a]	ICD-9 : 140-208<br>ICD-O-3: C00-C97
Hysterectomy	Defined from surgery performed[b]	NHI Procedure Code:<br>80403B、80404C、80411B、80412B、80413B、80414B、80416B、80421B、80429B、80430B、81005C、81029C、97022B、97027C、97037B、N26034、80026B、80027B、78020B、80418B、80417B、80424B、N26037
**Comorbidities**		
DM	Defined from diagnosis[c]	ICD-9 : 250
PCOS	Defined from diagnosis[d]	ICD-9 : 256.4
Obesity	Defined from diagnosis[d]	ICD-9 : 278.0
HPL	Defined from diagnosis[d]	ICD-9 : 272.4、272.9
**Drug Use**		
HRT	Defined from drug prescription[e]	ATC code : G03C、G03F
Statin use	Defined from drug prescription[e]	ATC code : C10AA、C10BX03

a Cancer including uterine cancer was established based on at least one time records of ICD-9 codes and ICD-O-3 in Taiwan cancer registry database. **b** The surgery performed was established based on at least one time record of procedure codes in the NHI inpatient database. **c** To ensure accuracy, DM were established based on at least three times inpatient or outpatient records of ICD-9 codes in the NHI database. **d** Comorbidities were established based on at least one time inpatient or outpatient records of ICD-9 codes in the NHI database. **e** Drug usage was established based on at least one time inpatient or outpatient record of drug prescription codes in the NHI database.

Table S2. Histopathological subtypes of uterine cancers according to the World Health Organization Classification of Tumors.

Clinical outcomes		
Uterine cancer	Defined from diagnosis[a]	ICD-9 : 182<br>ICD-10 : C54
Subtype of uterine cancer	-	-
Endometrioid	Defined from diagnosis[f]	ICD-O-3 M-code : 8140、8262、8380、8381、8382、8383、8480、8481、8570
Clear cell	Defined from diagnosis[f]	ICD-O-3 M-code : 8310
Serous	Defined from diagnosis[f]	ICD-O-3 M-code : 8461
Carcinosarcoma	Defined from diagnosis[f]	ICD-O-3 M-code : 8950、8980
ESS	Defined from diagnosis[f]	ICD-O-3 M-code : 8930
LGESS	Defined from diagnosis[f]	ICD-O-3 M-code : 8931
LMS	Defined from diagnosis[f]	ICD-O-3 M-code : 8890、8891、8896
Adenosarcoma	Defined from diagnosis[f]	ICD-O-3 M-code : 8933
Others	Defined from diagnosis[f]	Diagnosed with uterine cancer, but not classified as above

a Cancer including uterine cancer was established based on at least one time records of ICD-9 codes and ICD-O-3 in Taiwan cancer registry database. **f** The uterine cancer subtype was defined based on the ICD-O-3 morphological code in the database of the Taiwan Cancer Registry. **ICD-9**: International Classification of Diseases, 9th Revision, **ICD-O-3**: International Classification of Diseases for Oncology, 3rd edition, **NHI**: National Health Insurance, **M-code**: morphology code, **ESS** denotes endometrial stromal sarcoma, **LGESS** low grade endometrial stromal sarcoma, **LMS** leiomyosarcoma.

Table S3. Adjusted HR of uterine cancer in different subgroups stratified by HPL and statins in age < 50 years and ≥ 50 years.

		< 50 years				≥ 50 years			
HPL	Statins	N	Adjust HR	95% CI	P	N	Adjust HR	95% CI	P
–	–	3207338	ref	-	-	1297693	ref	-	-
–	+	33351	1.41	1.18 - 1.68	0.0001	97824	0.98	0.87 - 1.09	0.6567
+	–	119637	1.38	1.25 - 1.52	<.0001	155285	0.92	0.84 - 1.01	0.0795
+	+	43069	1.59	1.37 - 1.84	<.0001	150045	0.85	0.78 - 0.94	0.0012

Adjust variables: DM, PCOS, obesity, hyperlipidemia, HRT, HR denotes hazard ratio, CI confidence interval, HPL hyperlipidem.
